# Role of vitamin D receptor gene *Cdx2* and *Apa1* polymorphisms in prostate cancer susceptibility: a meta-analysis

**DOI:** 10.1186/s12885-016-2722-2

**Published:** 2016-08-23

**Authors:** Kewei Wang, Guosheng Wu, Jinping Li, Wentao Song

**Affiliations:** 1 Wuxi Medical School, Jiangnan University, Wuxi, Jiangsu 214122 People’s Republic of China; 2 Nanchang Center for Disease Control and Prevention, 833 Lijing Road, Nanchang, Jiangxi People’s Republic of China

## Abstract

**Background:**

Vitamin D receptor (*VDR*) gene polymorphisms affect the risk of prostate cancer. However, studies investigating the relationship between VDR gene polymorphisms (*Cdx2* and *ApaI*) and prostate cancer risk are equivocal. Therefore, we conducted a meta-analysis of all the studies to review the evidence available.

**Methods:**

A comprehensive search of PubMed, EMBASE, and ISI Web of Science for studies published until September 2015 was conducted. Odds ratios (ORs) and 95 % confidence intervals (CIs) were analyzed to determine the association between VDR *Cdx2* and *ApaI* polymorphisms, and prostate cancer risk.

**Results:**

The meta-analysis included 10 studies involving 4979 cases and 4380 controls to analyze the VDR *Cdx2* polymorphism. An additional 11 studies involving 2837 cases and 2884 controls were analyzed for the VDR *ApaI* polymorphism. Evidence failed to support the role of VDR *Cdx2* and *ApaI* polymorphisms in prostate cancer. For *Cdx2*, the pooled OR was 1.11 (95 % CI = 0.93–1.33) for AA vs. GG genotypes, 0.97 (95 % CI = 0.88–1.06) for GA vs. AA genotypes, 0.99 (95 % CI = 0.91–1.08) for AA + GA vs. GG, and 1.12 (95 % CI = 0.95–1.31) for AA vs. GA + GG. No significant relationship was observed in any subgroup analysis based on ethnicity, controls, and Hardy–Weinberg equilibrium (HWE). ORs for the ApaI polymorphism were similar.

**Conclusions:**

VDR *Cdx2* and *ApaI* polymorphisms are not associated with prostate cancer. Additional evidence is required to confirm this conclusion.

## Background

Prostate cancer ranks second among cancers diagnosed worldwide and sixth among cancer-related deaths in males. In 2012, more than 1.1 million cases were newly diagnosed worldwide. Prostate cancer accounts for 15 % of all cancers in men, and nearly 759,000 are reported in developed countries. In 2012, prostate cancer ranked fifth among cancer-related deaths in men, accounting for nearly 307,000 deaths or 6.6 % of all cancer-induced deaths in males [1]. Furthermore, the number of prostate cancers newly diagnosed annually is expected to climb to 1,853,391 worldwide by 2030, resulting in almost 544,209 deaths [2]. Studies suggest that ethnicity, diet, aging, and genetic factors mediate the pathophysiology of prostate cancer [3–5]. Therefore, the prevalence of prostate cancer among African-Americans, Caucasians, and Asians varies [6].

The role of genetics in prostate cancer has been the focus of research attention in recent years. *BRCA1* and *BRCA2* mutations increase the risk for ovarian and breast cancer as well as prostate cancer [7]. Hereditary prostate cancer gene 1 (*HPC1*), androgen and vitamin D receptors have been linked to prostate cancer [8]. Genome-wide association studies [9, 10] reported several SNPs substantially increasing the risk of prostate cancer.

The role of testosterone and vitamin D in prostate cancer is mediated via vitamin D receptor (VDR). The hormonally active form of vitamin D1, 25-dihydroxyvitamin D, inhibits cancer progression [11]. Vitamin D lowers the risk of several types of cancer, including prostate [12]. VDR is encoded by a large gene (>100 kb) mapped to chromosome 12q12-14. Its 14 exons spanning approximately 75 kb [13, 14] exhibit a high degree of polymorphism, with at least 618 reported variants, most of which are either undetectable or occur at a low frequency in the general population. Among the known VDR polymorphisms, the most common SNPs, influencing the VDR expression in prostate cancer include *FokI, BsmI, ApaI, Cdx2*, and *TaqI* [15–18]. However, these associations between SNPs and prostate cancer are not proven. The role of *BsmI*, *TaqI*, and *FokI* polymorphisms in prostate cancer is not established [19, 20]. Similarly, *ApaI* and *Cdx2* polymorphisms in prostate cancer risk are not validated [15, 16, 21–28]. For example, a case-controlled study showed a two-fold higher risk in Caucasian homozygous aa carriers for the variant *ApaI* compared with homozygous AA carriers [28]. Torkko reported that the *Cdx2* polymorphism significantly increased the prostate cancer risk among Hispanic populations carrying the SRD5A2 V89L VV genotype [27]. However, a study conducted by Rowland found no relationship between prostate cancer and *ApaI* and *Cdx2* SNPs [29].

The discrepancies may be attributed partly to statistical weakness, heterogeneity, population diversity, minimal effect of polymorphisms, and publication bias. We, therefore, investigated the role of VDR *Cdx2* and *ApaI* polymorphisms in prostate cancer risk by conducting a meta-analysis of all the eligible case-controlled studies.

## Methods

### Study selection

We searched PubMed, EMBASE, and ISI Web of Science databases for genetic association studies involving VDR *ApaI* and *Cdx2* polymorphisms and prostate cancer susceptibility, published through September 2015. We used combinations of the following keywords: ‘prostate cancer’, ‘VDR’ or ‘vitamin D receptor’, ‘ApaI’ or ‘rs7975232’, ‘Cdx2’ or ‘rs11568820’, and ‘polymorphism’, ‘variant’, or ‘mutation’. Two independent investigators (Kewei Wang and Guosheng Wu) performed the search. Additional articles were retrieved via manual searches of reference lists in the studies identified initially. Our search was not restricted by publication date or language. Selected articles are listed in Table 1 with the following data: the first author, publication year, country, ethnicity, source of controls, number of cases and controls, polymorphisms, and Hardy-Weinberg equilibrium (HWE) (*P* value). Other eligible studies were retrieved for additional review and data extraction. All the investigators were qualified and trained in literature search, statistical analysis, and evidence-based medicine.

**Table 1:** Characteristics of eligible studies

First author	Year	Country	Ethnicity	Total sample size (case/control)	Genotyping method	Source of control	Study	Polymorphisms	P for HWE
Gilbert [18]	2015	UK	Caucasian	951/898	Taqman	PB	NCC	Cdx2	0.96
				950/890	Taqman	PB	NCC	ApaI	0.09
Jingwi [19]	2015	USA	African American	446/379	TaqMan	PB	CC	Apa1	0.89
Yousaf [43]	2014	Pakistan	Asian	47/134	PCR-RFLP	PB	CC	Apa1	<0.0001
Jin Oh [41]	2014	Korean	Asian	272/173	PCR-RFLP	HB	CC	Cdx2	─
Rowland (A) [56]	2012	USA	African American	414/223	TaqMan	PB	CC	Cdx-2	0.07
			Caucasian	1117/795	TaqMan	PB	CC	Cdx-2	0.55
Bai [73]	2009	China	Asian	122/130	PCR-RFLP	HB	CC	ApaI	0.21
Onen [28]	2008	Turkey	Caucasian	133/157	PCR-RFLP	HB	CC	Apa1	0.41
Torkko [27]	2008	USA	Hispanic White	141/273	TaqMan	PB	CC	Cdx-2	0.05
			Caucasian	444/488	TaqMan	PB	CC	Cdx-2	0.99
Mikhak [26]	2007	USA	Caucasian	688/689	TaqMan	PB	CC	Cdx-2	0.15
Chaimuangraj [74]	2006	Thailand	Asian	28/74	PCR-RFLP	HB	CC	Apa1	0.88
Cicek [23]	2006	USA	Caucasian	439/479	TaqMan	PB	CC	Cdx2	0.26
				439/478	TaqMan	PB	CC	Apa1	0.25
John [24]	2005	USA	Caucasian	417/435	TaqMan	PB	CC	Cdx-2	0.75
Huang [15]	2004	Taiwan	Asian	160/205	PCR-RFLP	HB	CC	Apa1	0.03
Oakley-Girvan [25]	2004	USA	African American	113/121	PCR-RFLP	PB	CC	Apa1	0.16
			Caucasian	232/171	PCR-RFLP	PB	CC	Apa1	0.19
Maistro [42]	2004	Brazil	mixed	165/200	PCR-RFLP	PB	CC	Apa1	─
Bodiwala [22]	2004	UK	Caucasian	368/243	PCR-RFLP	HB	CC	Cdx-2	0.21
Suzuki [16]	2003	Japan	Asian	81/105	PCR-RFLP	PB	CC	Apa1	0.007
Habuchi [21]	2000	Japan	Asian	222/337	PCR-RFLP	HB	CC	Apa1	0.96

### Inclusion and exclusion criteria

The inclusion criteria were: (1) studies evaluating VDR *Cdx2* and *ApaI* polymorphisms and prostate cancer risk; (2) clinical studies; (3) case–control studies; (4) studies investigating diseases confirmed histologically, pathologically and/or radiologically; (5) adequate genotype distributions to facilitate estimation of OR with 95 % CI; and (6) most recent or complete studies. The exclusion criteria were:: (1) studies containing overlapping data; (2) missing genotype or allele frequencies; (3) absence of case controls; (4) studies not analyzing VDR *Cdx2* and *ApaI* polymorphisms in prostate cancer susceptibility; (5) studies investigating progression, severity, phenotype modification, response to treatment, or survival; (6) inadequate data extraction; or (7) missing genotype frequencies.

### Meta-analysis

ORs with 95 % CIs were used to measure the relationship between VDR *Cdx2* and *ApaI* polymorphisms, and prostate cancer risk. The Z test was used to evaluate the significance of pooled OR. *P* value less than 0.05 was deemed significant. Homozygote, heterozygote, recessive and dominant models were used to determine the association of *Cdx2* and *ApaI* polymorphisms with prostate cancer risk.

Statistical heterogeneity was evaluated using chi-square-based Q-statistic [30] and I^2^ statistic [31]. *P* < 0.10 or I^2^ > 50 % suggested statistically significant heterogeneity. A random effects model was used to calculate the pooled OR estimates. In other cases, a fixed effect model was used [32].

Sensitivity and subgroup analyses were used to explore the sources of heterogeneity among studies. Sequential exclusion of individual studies facilitated the evaluation of stability and sensitivity of the results. Subgroup analyses were based on ethnicity, controls and HWE.

Begg’s funnel plots were used to determine publication bias in studies. Linear regression asymmetry was tested using the procedure described by Egger et al. [33]. An asymmetric plot suggested possible publication bias. *P* value less than 0.05 in Egger’s test indicated significant publication bias.

The statistical tests were conducted using STATA statistical software (version 12.0 STATA Corp., College Station, TX). All *P* values were two-sided. The reliability and accuracy of the results were ensured by two authors independently evaluating the data with the same software.

## Results

### Eligible studies

The search terms returned 292 publications. We excluded 266 studies unrelated to Vitamin D receptor (VDR) gene polymorphism, three studies unrelated to prostate cancer [34–36], and three reviews [37–39]. The remaining 20 studies were included in the meta-analysis. We excluded two meta-analyses [20, 40], and two other studies [41, 42], which lacked genotype frequencies. No additional studies were retrieved following manual search of references in the published studies. Therefore, a total of 16 relevant studies were eligible for inclusion in the meta-analysis (Table 1). Three of the eligible studies reporting data involving two different ethnic groups were treated independently [25, 27]. Therefore, the final meta-analysis included a total of 19 case-controlled studies as shown in Table 1. Seven studies involved 4979 cases and 4380 controls related to VDR *Cdx2* polymorphism and prostate cancer risk, and 11 studies involved 2837 cases and 2884 controls related to VDR *ApaI* polymorphism.

The sample size ranged from 28 to 1117 individuals. Six of the eligible studies involved Caucasians and four were conducted in other ethnic groups to investigate VDR *Cdx2* polymorphism. VDR *ApaI* polymorphisms were investigated in Caucasians in four studies. Six studies involved Asians, and two involved African-Americans. Ten studies involved population samples, and six were hospital-based. PCR-RFLP and TaqMan assays were used to study the polymorphisms. The genotype distributions were not in HWE among the controls in two studies investigating VDR *Cdx2* [27] and VDR *ApaI* [15, 16, 43].

### Primary and subgroup analyses

**Fig. 1:**
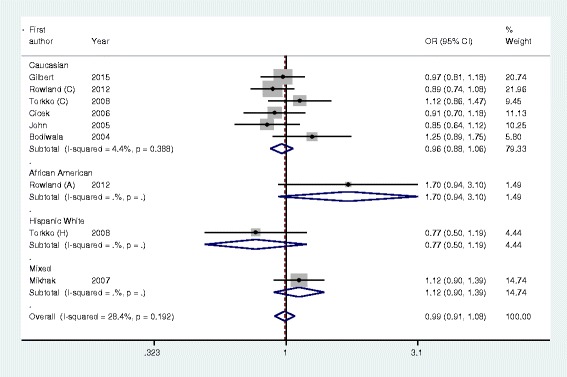
Forest plot of VDR Cdx2 polymorphism and prostate cancer risk using a fixed-effect model (dominant model AA + GA vs. GG)

As shown in Table 2, VDR *Cdx2* polymorphism was not significantly associated with prostate cancer risk in the pooled meta-analysis of all the eligible studies (homozygote model: AA vs. GG: OR = 1.11, 95 % CI = 0.93–1.33, *P* = 0.23; heterozygote model: GA vs. AA: OR = 0.97, 95 % CI = 0.88–1.06, *P* = 0.53; dominant model: AA + GA vs. GG: OR = 0.99, 95 % CI = 0.91–1.08, *P* = 0.80, Fig. 1; recessive model: AA vs. GA+ GG: OR = 1.12, 95 % CI = 0.95–1.31, *P* = 0.16). Subgroup analyses based on ethnicity, source of control, and HWE in controls, revealed no significant association.

**Table 2:** Meta-analysis of VDR Cdx2 polymorphism and prostate cancer risk

		Homozygote (AA vs. GG)			Heterozygote (GA vs. GG)			Dominant model (AA + GA vs. GG)			Recessive model (AA vs. GA + GG)		
Analysis	N	OR (95 % CI)	P	I^2^ (%)	OR (95 % CI)	P	I^2^ (%)	OR (95 % CI)	P	I^2^ (%)	OR (95 % CI)	P	I^2^ (%)
Overall	9	1.11 (0.93–1.33)	0.23	32.3	0.97 (0.88–1.06)	0.53	12.9	0.99 (0.91–1.08)	0.80	28.4	1.12 (0.95–1.31)	0.16	17.6
Ethnicity													
*Caucasian*	6	1.13 (0.92–1.39)	0.23	23.0	0.93 (0.84–1.03)	0.201	0	0.96 (0.88–1.06)	0.45	4.4	1.15 (0.94–1.41)	0.15	16.7
*African American*	1	1.80 (0.97–3.32)	0.06	—	1.54 (0.81–2.92)	0.18	—	1.70 (0.94–3.10)	0.08	—	1.26 (0.91–1.75)	0.16	—
*Hispanic White*	1	0.49 (0.17–1.36)	0.17	—	0.83 (0.52–1.31)	0.43	—	0.77 (0.50–1.19)	0.24	—	0.52 (0.18–1.43)	0.20	—
*Mixed*	1	0.94 (0.58–1.50)	0.80	—	1.15 (0.91–1.44)	0.22	—	1.12 (0.90–1.39)	0.31	—	0.89 (0.56–1.42)	0.64	—
Source of *control*													
*PB*	8	1.11 (0.92–1.33)	0.259	40.7	0.95 (0.86–1.04)	0.32	0	0.97 (0.89–1.06)	0.54	23.8	1.12 (0.95–1.32)	0.16	27.7
*HB*	1	1.11 (0.93–1.32)	0.686	—	1.26 (0.88–1.80)	0.19	—	1.25 (0.89–1.75)	0.19	—	1.07 (0.52–2.18)	0.85	—
*HWE in controls*													
*Yes*	8	1.15 (0.95–1.40)	0.133	34.3 %	0.97 (0.89–1.07)	0.65	22.1	0.99 (0.91–1.09)	0.99	34.6	1.15 (0.97–1.36)	0.09	20.2
*No*	1	0.87 (0.54–1.40)	0.58	—	0.91 (0.69–1.21)	0.53	—	0.90 (0.69–1.17)	0.46	—	0.90 (0.57–1.43)	0.67	—

*P P* values for Z test, *OR* odds ratio, *CI* confidence intervals, *HB* hospital–based studies, *PB* population-based studies, *HWE* Hardy–Weinberg equilibrium

**Fig. 2:**
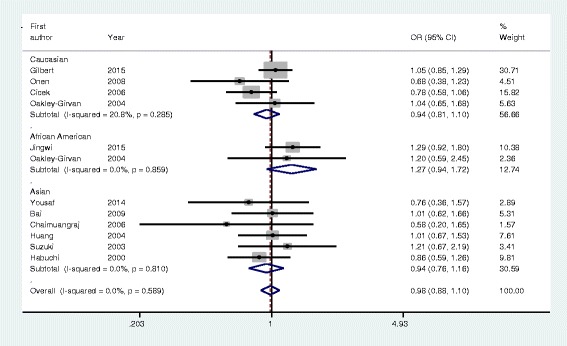
Forest plot of VDR ApaI polymorphism and prostate cancer risk using a fixed-effect model (dominant model AA + Aa vs. aa). OR, odds ratio; CI, confidence interval

As shown in Table 3, VDR *ApaI* polymorphism was not significantly correlated with prostate cancer risk in pooled analysis of eligible studies (homozygote model: AA vs. aa, OR = 0.97, 95 % CI: 0.76–1.25, *P* = 0.85; heterozygote model: Aa vs. aa: OR = 1.00, 95 % CI: 0.88–1.13, *P* = 0.99; dominant model: AA + Aa vs. aa: OR = 0.98, 95 % CI: 0.87–1.10, *P* = 0.79, Fig. 2; recessive model: AA vs. Aa + aa: OR = 0.97, 95 % CI: 0.85–1.01, *P* = 0.64). Subgroup analyses based on ethnicity, source of controls, and HWE in controls, revealed the absence of prostate cancer risk with VDR *ApaI* polymorphism.

**Table 3:** Meta-analysis of VDR ApaI polymorphism and prostate cancer risk

		Homozygote (AA vs. aa)			Heterozygote (Aa vs. aa)			Dominant model (AA + Aa vs. aa)			Recessive model (AA vs. Aa + aa)		
Analysis	N	OR (95 % CI)	P	I^2^ (%)	OR (95 % CI)	P	I^2^ (%)	OR (95 % CI)	P	I^2^ (%)	OR (95 % CI)	P	I^2^ (%)
Overall	9	0.97 (0.76–1.25)	0.85	51.1	1.00 (0.88–1.13)	0.99	28.8	0.98 (0.87–1.10)	0.79	0	0.97 (0.85–1.01)	0.64	0
Ethnicity													
*Caucasian*	4	0.81 (0.66–1.01)	0.06	11.0	1.01 (0.85–1.19)	0.91	20.8	0.94 (0.81–1.10)	0.46	20.8	0.92 (0.78–1.06)	0.25	0
*African American*	2	1.54 (0.74–3.19)	0.24	57.9	1.16 (0.84–1.60)	0.35	0	1.27 (0.94–1.72)	0.12	0	0.92 (0.65–1.29)	0.63	0
*Asian*	6	1.05 (0.69–1.58)	0.82	36.2	0.90 (0.71–1.14)	0.40	49.0	0.93 (0.76–1.16)	0.56	0	1.24 (0.92–1.66)	0.14	0
Source of *control*													
*PB*	8	1.03 (0.77–1.38)	0.82	60.1	1.05 (0.91–1.20)	0.52	42.9	1.02 (0.90–1.16)	0.72	0	0.97 (0.85–1.11)	0.64	17.8
*HB*	4	0.82 (0.50–1.34)	0.43	29.3	0.83 (0.63–1.10)	0.20	0	0.84 (0.65–1.09)	0.18	0	0.98 (0.69–1.38)	0.92	0
*HWE in controls*													
*Yes*	10	0.96 (0.76–1.21)	0.733	36.6	1.01 (0.89–1.15)	0.90	0	0.99 (0.87–1.12)	0.86	0	0.93 (0.82–1.06)	0.29	0
*No*	2	0.86 (0.19–3.86)	0.84	86.7	0.91 (0.59–1.40)	0.66	85.6	0.98 (0.88–1.10)	0.74	0	1.56 (0.99–2.46)	0.05	0

*P P* values for Z test, *OR* odds ratio, *CI* confidence intervals, *HB* hospital–based studies, *PB* population-based studies, *HWE* Hardy–Weinberg equilibrium, *NR* not reported

### Heterogeneity analysis and sensitivity analysis

Significant heterogeneity was found in AA vs. Aa genetic model of VDR *ApaI* polymorphism (*P* = 0.021, I^2^ = 51.1 %). Sensitivity analysis was conducted by excluding individual studies to determine heterogeneity. Sequential exclusion of individual case-controlled study revealed similar results statistically, indicating the stability and sensitivity of the meta-analysis (data not shown).

Population and subgroup analysis revealed no significant heterogeneity in terms of VDR *Cdx2* polymorphism.

### Publication bias

**Fig. 3:**
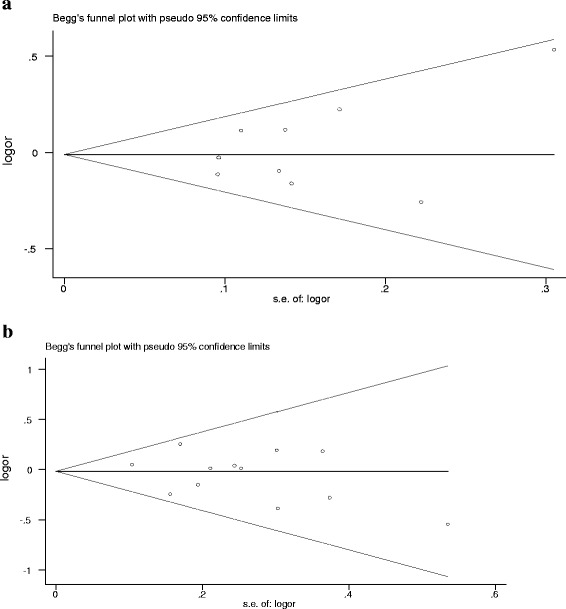
Funnel plot analysis for detection of publication bias. Each point represents a separate study for the indicated association. **a** Funnel plot: dominant model AA + GA vs. GG of VDR Cdx2 polymorphism in overall analysis (*P* = 0.67) and (**b**) Funnel plot: dominant model AA + Aa vs. aa of VDR ApaI polymorphism in overall analysis (*P* = 0.48)

Symmetrical Begg’s funnel plots indicated the absence of publication bias in the overall meta-analysis (Fig. 3). Egger’s test results revealed no publication bias in studies investigatingVDR *Cdx2* polymorphism (*P* = 0.67 for AA vs. GG; *P* = 0.24 for GA vs. GG; *P* = 0.34 for dominant model AA + GA vs. GG; and *P* = 0.248 for recessive model AA vs. GA + GG) and VDR *ApaI* (*P* = 0.80 for AA vs. aa; *P* = 0.78 for Aa vs. aa; *P* = 0.48 for dominant model AA + Aa vs. aa; and *P* = 0.14 for recessive model AA vs. Aa + aa).

## Discussion

Genetic polymorphisms play a key role in the pathophysiology of disease. Genome-wide association studies (GWAS) reported more than 90 common SNPs (minor allele frequency [MAF], 5 % or greater) with established relationship involving insignificant alterations (average per allele odds ratios [ORs]:1.1–1.3) in prostate cancer susceptibility [44–47]. Overall, the SNPs account for a third of the total inherited risk of prostate cancer [44, 45].

VDR is a nuclear receptor regulating bone mineral homeostasis, mammalian hair cycle, and compound detoxification. It has recently been found to prevent tumorigenesis by inhibiting cell proliferation and differentiation, and inducing apoptosis. Previous studies demonstrated that VDR gene polymorphisms, which include *FokI, BsmI, ApaI, TaqI,* and *Cdx2*, are associated with ovarian [48], skin [49], breast [50], and colorectal cancers [51].

The G-to-A polymorphism involving a Cdx2-binding site in the 1e promoter region, mediates *VDR* transcription in intestine [52]. The strong binding of A allele with the Cdx2 transcription factor enhances transcriptional activity [53]. Thus, Cdx2 regulates cellular proliferation and differentiation. The A allele frequencies varied in different ethnic groups: 74 % in Africans, 43 % in Asians, and 19 % in Caucasians [54].

*Cdx2* polymorphism prevents osteoporosis [53]. The *ApaI* polymorphisms (in intron 8) at the 3′ untranslated region (UTR) are in strong linkage disequilibrium (LD) [54]. Nonetheless, the polymorphism does not alter the predicted amino acid sequence of the VDR, and often affects mRNA stability and the efficiency of protein translation [55]. Several studies investigated the role of VDR *Cdx2* and *ApaI* polymorphisms in prostate cancer risk, with inconclusive results. Therefore, we conducted a meta-analysis to establish the association between VDR *Cdx2* and *ApaI* polymorphisms, and prostate cancer risk.

Our meta-analysis, including 6427 cases and 6039 controls from 16 case-controlled studies, evaluated the association between *Cdx2* and *ApaI* polymorphisms, and prostate cancer risk. Our results suggest that these polymorphisms do not increase the risk of prostate risk in genetic models, which was consistent with a previous meta-analysis [20]. However, our current meta-analysis included 6427 cases and 6039 controls from 16 case-controlled studies to obtain comprehensive results. Subgroup analysis based on ethnicity, source of control, and HWE in controls, showed no significant relationship between *Cdx2* and *ApaI* polymorphisms, and prostate cancer risk in any comparative studies.

The role of VDR *Cdx2* and *ApaI* polymorphisms in prostate cancer was unclear due to ethnic variation in genotypes, controls and subjects, and genotyping techniques [16]. The VDR *Cdx2* AA genotype is most frequently found in African-Americans (58.9 %) [56], while the GG genotype occurs most frequently in Hispanic Whites (65.7 %) [27].

The VDR *ApaI* genotype AA is the most prevalent in African-Americans (40.2 %) [25], while the aa genotype is most frequently found in Asians (67.9 %) [43]. However, the African study sample included three studies involving African-Americans, preventing statistical interpretation with confidence. A larger sample size is needed for subgroup analysis of various ethnic populations.

Furthermore, a few hospital-based studies did not support the association of increased risk with VDR polymorphisms compared with normal controls [57, 58], in contrast to other investigations [21, 59]. In subgroup analyses by source of control, we selected 16 studies (eight studies related to VDR *Cdx2* polymorphism and eight involving VDR *ApaI* polymorphism) which included subjects from more representative populations to determine potential gene association in tumorigenesis.

The relationship between VDR *Cdx2* and *ApaI* polymorphisms, and prostate cancer risk in previous studies is attributed to differences in lifestyle and disease prevalence as well as limited sample size [60–62]. Further, prostate size, cancer stage, and depth of invasion were not considered, in determining the genotypic distribution. Prostate cancer is a complex and multifactorial disease mediated by genetic and environmental factors in different populations [60].

However, the risk factors underlying prostate cancer are related to each other. Similar gene polymorphisms may still result in different phenotypes, because the penetrance of the mutation depends on the interaction with other polymorphisms and exposure to specific environment.

Genetic heterogeneity in meta-analysis of studies investigating genetic polymorphisms and various diseases is not surprising. However, no heterogeneity was observed among studies investigating the VDR *Cdx2* polymorphism in our meta-analysis. Different genotype distributions and population stratification may also alter genotype-phenotype associations.

Furthermore, a number of factors affect heterogeneity. Different studies select subjects for control groups based on different definitions, resulting in heterogeneity observed in our meta-analysis. We investigated whether the heterogeneity might be explained by potential confounding factors such as age, smoking, drinking, androgen levels, and other clinical characteristics. However, no reliable results were available due to lack of access to individual data involving these variables. Similar heterogeneity was observed with the VDR *ApaI* polymorphism.

Cancer is a complex disease, and is triggered by genetic factors as well as environmental impact (UV exposure), gene interactions, and lifestyle (e.g., smoking, drinking alcohol, and diet) [63–71]. Interaction between environmental factors and VDR gene is also a possibility [70, 72]. Further large studies investigating the different types of VDR *Cdx2* and *ApaI* polymorphisms are needed to facilitate subgroup analyses. Environmental interaction with VDR *Cdx2* and *ApaI* polymorphisms and its role in prostate cancer risk needs to be validated.

The study limitations of our meta-analysis are as follows. First, in subgroup analyses based on ethnicity, the population sample size was comparatively small, which may affect the statistical power in determining the significance of the relationship. Second, our results were not adjusted for variables such as age, smoking, drinking, obesity, gene-gene interactions, and environmental factors, due to lack of access to the original study data. Finally, most studies investigating the VDR *Cdx2* polymorphism in prostate cancer risk involved Caucasian population. Therefore, evidence based on large controlled studies involving a wide range of ethnic and population groups is needed to re-evaluate the association between specific SNPs and prostate cancer risk.

## Conclusions

Our findings suggest that VDR *Cdx2* and *ApaI* polymorphisms are not linked to prostate cancer susceptibility in the overall population. Epidemiological studies with large sample sizes including a wide range of ethnic populations and functional parameters are needed to reinforce our findings.

## Abbreviations

VDR: Vitamin D receptor
HPC1: Hereditary prostate cancer gene 1
HWE: Hardy–Weinberg equilibrium
PCR-RFLP: Polymerase chain reaction - restriction fragment length polymorphism
SNP: Single nucleotide polymorphism
OR: Odds ratio
CI: Confidence interval
HB: Hospital–based studies
PB: Population-based studies
